# Characterization of the Prokaryotic Sodium Channel Na_v_Sp Pore with a Microfluidic Bilayer Platform

**DOI:** 10.1371/journal.pone.0131286

**Published:** 2015-07-06

**Authors:** Shimul Chandra Saha, Alexander J. Henderson, Andrew M. Powl, B. A. Wallace, Maurits R. R. de Planque, Hywel Morgan

**Affiliations:** 1 Electronics and Computer Science, University of Southampton, Southampton, SO17 1BJ, United Kingdom; 2 Institute for Life Sciences, University of Southampton, Southampton, SO17 1BJ, United Kingdom; 3 Institute of Structural and Molecular Biology, Birkbeck College, University of London, London, WC1E 7HX, United Kingdom; Xuzhou Medical College, CHINA

## Abstract

This paper describes the use of a newly-developed micro-chip bilayer platform to examine the electrophysiological properties of the prokaryotic voltage-gated sodium channel pore (Na_v_Sp) from *Silicibacter pomeroyi*. The platform allows up to 6 bilayers to be analysed simultaneously. Proteoliposomes were incorporated into suspended lipid bilayers formed within the microfluidic bilayer chips. The chips provide access to bilayers from either side, enabling the fast and controlled titration of compounds. Dose-dependent modulation of the opening probability by the channel blocking drug nifedipine was measured and its IC_50_ determined.

## Introduction

Human voltage-gated sodium channels (Na_v_^'^s) are large pseudotetrameric integral membrane proteins which play important roles in many physiological processes and are linked to channelopathies including epilepsy, pain disorders and cardiac conditions such as long QT syndrome and Brugada syndrome [1–3]. Consequently they are major targets for drug development, and hence are of considerable interest to the pharmaceutical industry. Recombinant expression and purification of human channels have proved difficult due to their size and complexity in terms of post translational modifications. Prokaryotic voltage-gated sodium channels are simpler, single domain tetrameric orthologues which are more easily overexpressed, purified and reconstituted into lipid bilayers. Each monomeric subunit consists of 6 transmembrane helices (S1-S6), with S1-S4 comprising the voltage-sensing region and S5-S6 the pore region. Here we have expressed the isolated tetrameric pore-only construct of the prokaryotic voltage-gated sodium channel Na_v_Sp from *Silicibacter pomeroyi*. This pore-only construct has previously been shown to be correctly folded, more thermally stable than the full length channel and capable of supporting sodium flux, and is inhibitable by known eukaryotic channel blockers [4, 5]. Previously patch clamp techniques have demonstrated ensemble averaged currents for NaChBac, another voltage gated sodium channel orthologue, this time from *Bacillus halodurans*. Its activity is capable of being modulated by the calcium channel blocker drugs nifedipine, nimodipine and mibefradil [6]. Other orthologues such as the Na_v_Ms channel from *Magnetococcus marinus* [7] and the Na_v_Ab channel from *Arcobacter butzleri* [8] have been characterised by patch clamping of those channels expressed in HEK293 cells. Single channel characterisations of the Na_v_Sp pore and NaChBac have been performed using bilayer lipid membranes [5, 9], whilst sodium flux measurements of the Na_v_Sp pore and other channels and pore-only constructs [10] have been done using reconstituted proteins in liposomes. However to date, there is no data on the IC_50_ for any of these proteins at the single channel level when reconstituted into artificial lipid bilayer membranes.

**Fig 1:**
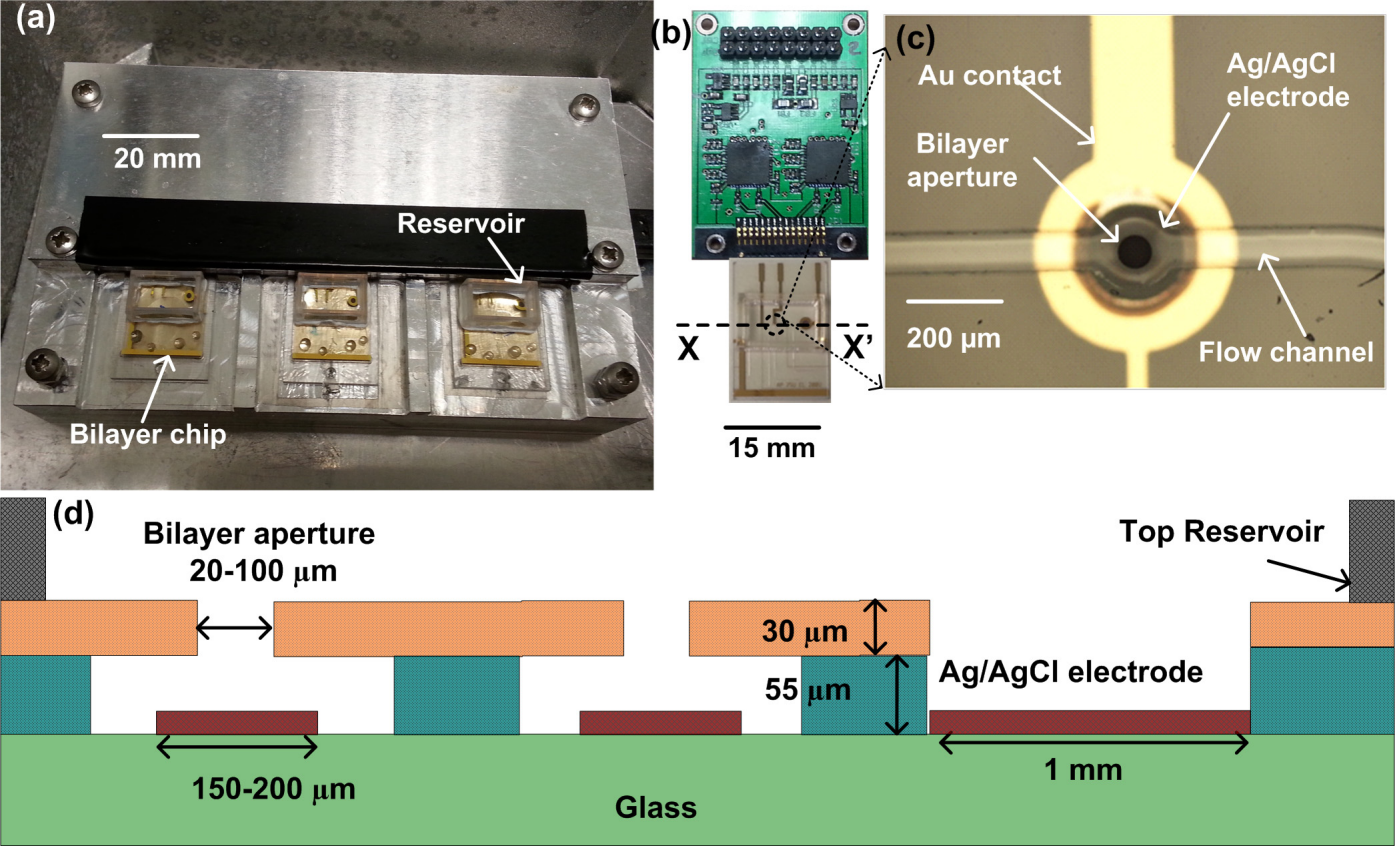
Bilayer platform for parallel channel recording. (a) Photograph of the BLM recording platform with three bilayer chips, each with two bilayers. (b) A daughter board showing two dual core ASIC amplifiers interfacing with the microfluidic chips. The integrated platform comprises three identical daughter boards, each of which can record from up to two bilayers, together with signal processing electronics. Data is transmitted to a computer via a USB connection. (c) Close up view of the bilayer aperture with Ag/AgCl electrode and flow channel. (d) Cross section X-X’ of the chip showing the dry film resist that forms the aperture together with the integrated Ag/AgCl electrodes, one common and one for each bilayer.

In this study, we use a miniature microfluidic ion-channel screening platform to characterise the Na_v_Sp pore construct. Bilayers were made across cavities fabricated in a microfabricated polymer support made on small glass chips. Each of these chips holds two independent bilayers and a miniature platform has been developed to accommodate three chips, allowing six bilayers to be continuously measured in parallel, as shown in Fig 1(A). The microfluidic chips interface with custom-designed integrated amplifiers (ASICs) and recording hardware/software as shown in Fig 1(B). The glass chips contain integrated Ag/AgCl electrodes together with edge connectors that make direct contact to the ASICs, significantly reducing contact resistance and noise. The bilayers are formed across miniature apertures made in an epoxy photoresist and the design provides fluid access on both sides, via a microfluidic channel (Fig 1C and 1D) that provides a simple means of quickly introducing compounds to the bilayer using passive pumping from droplets, eliminating syringe pumps and associated tubing [11]. In this work, the ion channels are inserted into the bilayer by the addition of proteoliposomes which spontaneously fuse with the lipid bilayer, leading to incorporation of the pores. To increase the probability of fusion the bilayers were formed across relatively large apertures, with a diameter of 75 μm. For the Na_v_Sp pore, spontaneous vesicle fusion and incorporation of active protein was infrequent although channel incorporation was facilitated, to some extent, by breaking and reforming the bilayers in the presence of proteoliposomes. However, the simultaneous acquisition of data from all 6 bilayers significantly increased the probability of observing active channels. Single-channel current and channel open probabilities were determined in the presence of the channel blocker nifedipine, enabling the determination of IC_50_ curves for this drug.

## Materials and Methods

### Materials

Lipids were purchased from Avanti Polar Lipids (Alabaster, AL, USA). N-dodecyl-β-D-maltopyranoside (DDM) and 5-cyclohexyl-1-pentyl-β-D-maltoside (Cymal-5) were obtained from Anatrace (Maumee, OH, USA). Solvents and nifedipine were from Sigma-Aldrich (Gillingham, UK) and Biobeads SM-2 from Bio-Rad (Hemel Hempstead, UK).

The Na_v_Sp pore-only construct was expressed and purified as previously described [10], with the following modifications: 26 litres of cultures were grown at 37°C for 3 hours after induction by 300 μM isopropyl-β-D-thiogalactopyranoside. The detergent was exchanged from 1% (w/v) n-dodecyl-β-D-maltopyranoside (DDM) to 0.52% HEGA10 during the elution of the protein from the HisTrap column. Additional purification of the tetrameric channel was achieved by gel filtration using a Superdex 200 Increase 10/300 GL column.

### Protein reconstitution into proteoliposomes

Chloroform solutions of the lipids 1-palmitoyl-2-oleoyl-*sn*-glycero-3-phosphoethanolamine (POPE) and 1-palmitoyl-2-oleoyl-*sn*-glycero-3-phospho-(1'-*rac*-glycerol) (POPG) were mixed at a molar ratio of 1:1 in glass scintillation vials and the chloroform was removed via evaporation using a nitrogen stream. The lipid film was then resuspended in 1 ml reconstitution buffer (20 mM Tris, 150 mM NaCl and 0.25% (w/v) Cymal 5). After sonication in a water bath for 15 minutes purified protein was added at a concentration of 17 mg/ml. Depending on the volume of protein-detergent solution, a protein to lipid ratio of 300:1, 600:1, 1000:1 or 3000:1 was obtained. Subsequently Biobeads were added to each vial to remove the detergent from the solution, with concomitant formation of proteoliposomes. After one hour incubation at room temperature, the proteoliposome dispersion was carefully removed from the vial with the Biobeads, flash frozen in 50 μl aliquots and stored at -80°C.

### Device design and fabrication

Fig 1(A) shows the platform with three microfluidic chips. Fig 1B and 1D) is a photograph and diagram of the disposable glass chip showing the microfluidic channel, the micro-aperture where the bilayer is formed and the integrated recording electrodes. The chip is made from a standard glass substrate (700 μm thick) and laminated dry film resist to form the aperture; full fabrication details have been published elsewhere [12]. The chip interfaces with custom designed ASIC (application specific integrated circuit) amplifiers, with each chip enabling simultaneous electrical recording from two bilayers [12,13]. Fig 1(B) also shows a photograph of a printed circuit board with two ASICs interfacing with a glass microfluidic chip. Each microfluidic chip contains two bilayers with microfluidic flow channels providing independent access to the bottom side of the bilayer. These flow channels are 100 μm wide and 55 μm high. Buffer on the cis side, i.e. above the bilayer, is retained within a plastic reservoir of 200 μl volume. Liquid is driven beneath the bilayers using passive pumping [14]. In this technique, buffer flows from a small droplet placed on the pumping port to a larger droplet on the reservoir port due to differences in Laplace pressure between the two different sized droplets. Fig 1(C) is a photograph of the bilayer aperture with the integrated Ag/AgCl electrode and the flow channel. Bilayers were made by painting lipid across the aperture and are stable for flow rates up to 0.4 μl/min [11]. Compared with traditional bilayer electrophysiology systems, both the bilayer capacitance and shunt capacitance is reduced so that higher bandwidth, low noise electrical recordings are possible [12].

### Bilayer measurements

Bilayers were formed as follows: the top compartment of the chamber was filled with electrophysiology buffer (0.5 M NaCl, 10 mM HEPES, pH 7.4) and a slight positive pressure was applied to fill the microfluidic channel. A 2.5 μl drop of buffer was placed at one end of the microfluidic channel, and an 8 μl drop at the other end to initiate flow. Bilayers were made by painting 3–5 μl of a 20 mg/ml solution of lipid (POPE/POPG, 1:1 molar ratio) in decane over an aperture with a brush of acrylic fiber. The lipid-decane solution was collected in a micro-pipette tip (10 μl volume) and released onto the brush tip (size 4/0) before painting. Bilayer formation was monitored by capacitance measurements; bilayers were formed at the first attempt approximately 90% of the time, while repainting enabled a 100% success rate. For a 75 μm diameter aperture with a suspended lipid bilayer, the capacitance was typically 15–20 pF, against a background capacitance of ~2–3 pF. All measurements were performed at room temperature.

### Data analysis

Ion channel currents were recorded using a custom data acquisition software interface [13] and then exported to Clampfit electrophysiology software [15] for analysis. All electrophysiological measurements were obtained using the ASIC system with an over-sampling frequency of 1.25 MHz. Data was subsequently digitally low-pass filtered at 1.25 kHz. The single-channel amplitude was determined from current histograms and by direct inspection of the current amplitude. The channel opening probability was calculated as the ratio of summed opening times to the total time of a selected trace. The channel is assumed open when the current amplitude is higher than half of the amplitude at the specific voltage. The opening probability for each drug concentration was normalized to a control condition (without any drug) and plotted against the drug concentration. The data was fitted to the Hill equation using Sigmaplot [16], and the half inhibitory concentration (IC_50_) was calculated as the concentration when the open probability is 50% of the control condition.

## Results and Discussion

### Ion channel recordings and current-voltage relationship

**Fig 2:**
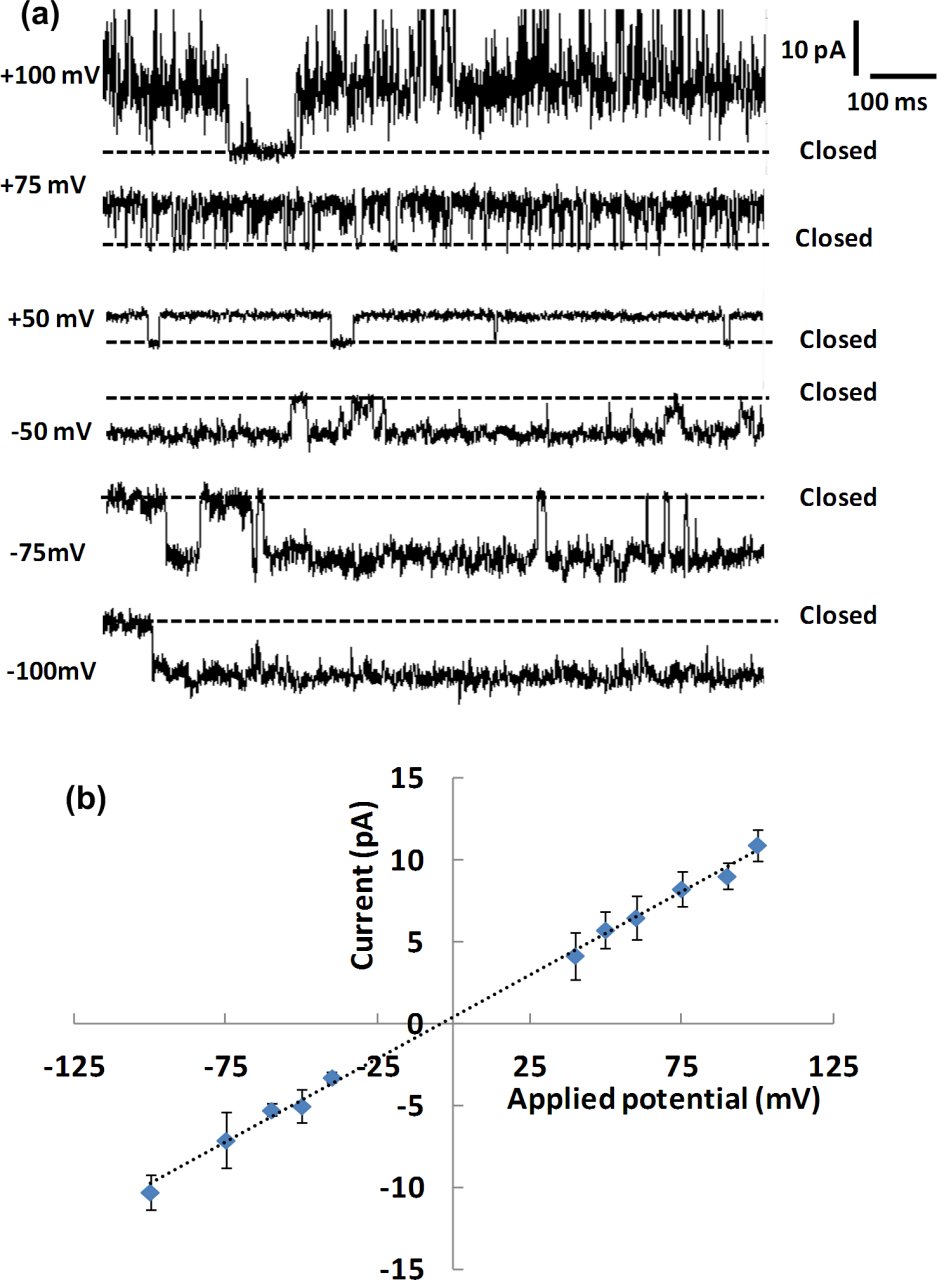
Ion channel events and their current voltage relationship. (a) Representative single channel current traces for the Na_v_Sp pore at different applied transmembrane potentials. (b) I-V plot for the Na_v_Sp pore. Error bars are one standard deviation for ≥ 3 individual bilayer measurements. The electrophysiology buffer is 0.5 M NaCl, 10 mM HEPES, pH 7.4. Channels were introduced in the aperture-suspended bilayer of POPE:POPG (1:1) by fusion of proteoliposomes with a lipid-to-protein ratio of 300:1. Data were recorded at a sampling frequency of 1.25 MHz and were low pass-filtered at 1.25 kHz.

Bilayers were made using POPC and POPG lipids since previous ion flux studies for these pore-only constructs [10] showed that this lipid composition is suitable for observing active channels. Successful recordings were most frequently obtained from liposomes with 300:1 lipid-to-protein ratios. For a symmetrical salt concentration (0.5 M NaCl, 10 mM HEPES, pH 7.4), typical single channel events are shown in Fig 2(A). At this NaCl concentration, the single channel conductance was 106 pS. I-V plots are shown in Fig 2(B); no rectifying behaviour was observed. This is not unexpected since the pore-only construct does not contain the voltage sensor region of the channel. The electrical behaviour is generally similar to data from Shaya *et al*. [5] for the Na_v_Sp1 pore-only construct, inserted into giant unilamellar vesicles (GUVs) made of 1,2-diphytanoyl-*sn*-glycero-3-phosphocholine (DPhPC) for planar patch clamp electrophysiology. The authors used asymmetric salt conditions (200 mM NaCl and 110 mM KCl) and measured channel conductances of ~40 pS. Full length NaChBac channels have also been characterized using both whole cell patch clamp [6] and bilayer lipid membranes [9]. The patch clamp method gave a channel conductance of 12 pS (8 mM and 130 mM NaCl), which is much less than determined from our data, but the lipid bilayer studies yielded conductance values of 120 pS for symmetric salt conditions (150 mM NaCl), very close to the values measured in this study for the pore. Our data indicates that Na_v_Sp is mostly open when inserted into the bilayer, but the opening time and open probability varies from experiment to experiment. This behaviour has also been observed by others, both for pore-only constructs [5] and for full length channels [6, 9].

### Interaction with nifedipine

**Fig 3:**
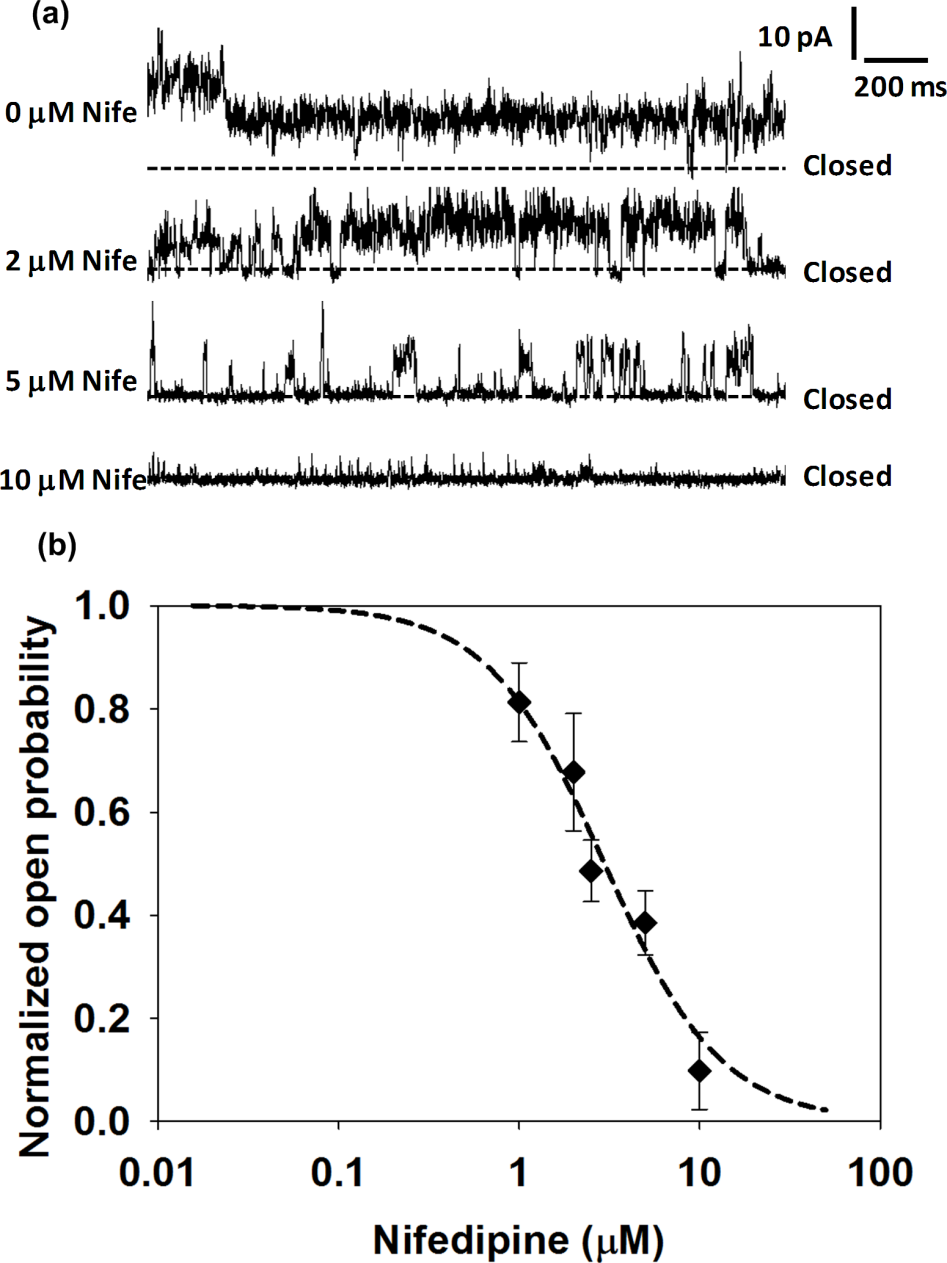
Modulation of ion channel properties by drugs. (a) Single channel current recordings of the Na_v_Sp pore for different concentration of nifedipine. (b) Channel open probability at different concentrations of the drug. Error bars are the standard deviations of measurements from ≥ 2 individual bilayers.

The calcium- and sodium-channel blocker nifedipine is used to treat high blood pressure and to control angina [17,18]. Nifedipine has been shown to reduce channel opening times and block prokaryotic sodium channels such as NaChBac [6] as well as eukaryotic calcium channels. Nifedipine was used in this study in preference to other channel blockers such as mibefradil due to its higher solubility in aqueous solutions. Upon addition of nifedipine to only one side of the membrane, complete blocking was unachievable even at very high concentrations, implying that the channels probably insert in both orientations so that the drug is unable to reach the binding sites of a large proportion of channels. Therefore, the half inhibitory concentration IC_50_ was measured by adding different concentrations of drug to both sides of the bilayer. Drugs were added after characteristic channel activity was established, since the gating behaviour (the mean open time) can vary from experiment to experiment. The open time probability was normalized against opening times for the channel in the absence of nifedipine. The data is plotted in Fig 3 and the IC_50_ was calculated to be 2.95 μM.

Complete block of the Na_v_Sp pore in bilayers, has been reported at concentrations of 100 μM for, the channel blocker mibefradil [5], whereas ~50% block of the sodium flux in liposomes was reported for the same pore at the same concentration of drug [4]. The only reported the IC_50_ value for nifidepine on prokaryotic sodium channels is by Ren *et al*. [6], using whole cell patch clamp methods. The authors investigated modulation of ensemble-averaged currents for full length (i.e. with voltage sensors) NaChBac and obtained an IC_50_ value of 2.2 μM, very similar to the value for the pore-only Na_v_Sp construct measured in this study.

## Conclusions

In summary, a novel multi-bilayer platform has been used to investigate the conductance and drug binding kinetics of a purified reconstituted prokaryotic sodium channel pore. The properties of the Na_v_Sp pore were characterised using a microfluidic platform that records from 6 bilayers simultaneously. The use of multiple bilayers increases the overall probability of obtaining recordings from single channels and thus the throughput of experiments. Single channel electrophysiological recordings of the Na_v_Sp pore showed an absence of rectification, as expected for constructs without the voltage sensor regions. The modulation of channel current (opening probability) by nifedipine was measured to determine the half inhibitory concentration of the drug. Functional studies of drug binding to the Na_v_Ms bacterial sodium channel expressed in HEK cells (in conjunction with crystallographic characterisation of their binding sites) has recently shown that the affinities of these channels for many drugs correspond closely to those for human Na_v_1.1 sodium channels [19]. Here we have explored the relationship between a prokaryotic channel pore and a commonly used human channel blocker in purified, reconstituted channels. This suggests that using such a multi-bilayer platform could further aid in screening potential drug candidates and could aid in understanding of the mode of action of channel blockers on voltage-gated sodium channels.
